# Paternal Role of Fathers in Families of Parents With Intellectual Disabilities: Views, Barriers and Facilitators in Fulfilling This Role

**DOI:** 10.1111/jar.70090

**Published:** 2025-06-27

**Authors:** M. van Nieuwenhuijzen, P. Riksten, S. Koet, M. Lever

**Affiliations:** ^1^ Research Institute Child Development and Education University of Amsterdam Amsterdam the Netherlands; ^2^ Expect Jeugd Partners voor Jeugd Amsterdam the Netherlands; ^3^ TOP Groep Zevenaar the Netherlands

**Keywords:** barriers and facilitators, fathers, parenting support, parents with intellectual disabilities, paternal role, professionals' views

## Abstract

**Background:**

This study examined the paternal role of fathers from families headed by parents with intellectual disabilities, their views and their barriers and facilitators in fulfilling this role.

**Method:**

Nine fathers of families headed by parents with intellectual disabilities and 14 professionals were interviewed. Transcriptions were coded using a framework analysis.

**Results:**

Fathers find their paternal role important and want to fulfil it by providing for the family, doing activities and teaching their child. However, they are hampered in fulfilling their paternal role due to several barriers at the family, professional and societal levels. Facilitators are co‐parenting, recognising fathers in parenting support, working relations and supporting parents in roles and rights.

**Conclusion:**

The awareness of the importance of the paternal role in parenting should increase. Professionals should support both mothers and fathers in fulfilling their parental roles to improve parental rights and the development and well‐being of their children.

**Summary**

- Fathers are important for the development of the child.
- But, fathers of families headed by parents with intellectual disabilities often experience barriers in fulfilling their paternal role because of problematic relationships with the mother of the child, not being involved in parenting support by professionals and stigmatisation by society.
- In case of a problematic relationship with the other parent or separation, parents have a hard time to coparent. Fathers may be less available for their children when they live somewhere else, work or when they have personal problems. In addition, social services omit to inform and involve fathers in parenting support, and fathers feel stigmatised by professionals and society.
- Fathers should be involved in parenting support; they should be informed and given attention by social services as well. Both parents should be supported in fulfilling their parenting roles and claiming their legal rights.

## 1 Introduction

Over the last decades, more attention has been given to parents and parenting with intellectual disabilities. Parenting can be an extra challenge for parents with intellectual disabilities because they often face a cumulation of complex problems (Meppelder et al. 2015; Wilson et al. 2013), such as mental health problems, poverty and traumatic experiences in their own youth (Emerson et al. 2015; Hindmarsh et al. 2015; McGaw et al. 2007; Powell et al. 2017). In addition, stigma and denial of fundamental rights of people with intellectual disabilities still exist in society (Scior et al. 2020) and higher (digital) demands of society hampers inclusion (Tatsou 2021). Nevertheless, some parents with intellectual disabilities are capable of raising their children, and some can parent when given adequate support in line with their needs (Collings and Llewellyn 2012; IASSID 2008; Koolen et al. 2020; Zijlstra et al. 2023).

However, the support and interventions for families of parents with intellectual disabilities are often mainly focused on mothers while the fathers of these families are hardly, or not at all, involved (Symonds et al. 2020). Nevertheless, it has been established that fathers in general play an important role in parenting and positively influence the development of their children. Involvement of the father can reduce stress during adolescence, reduce behavioural problems, improve language and cognitive development and stimulate more positive relationships with peers for their children (Cabrera et al. 2000; Pleck 2012). In addition, fathers can reduce stress for mothers by supporting them in parenting, especially when parenting is more challenging (Booth and Booth 2002; Boyd et al. 2019; Hartley et al. 2011; Kanter and Proulx 2019; Malinen et al. 2017).

Despite the important role of fathers, they have often been overlooked and hardly included in research on parenting (Cabrera et al. 2018), let alone in families headed by parents with intellectual disabilities. As a consequence, information is lacking on fathers' experience of their paternal role and their needs towards professionals and the support system. The goal of this study, therefore, is to gain more knowledge on the views of fathers from families headed by parents with intellectual disabilities on their paternal role and how parenting support can be more attuned with the fathers' needs to fulfil this role.

Although in Western countries and cultures fathers in general were seen primarily as a financial provider and authority figures in the past, today a different and broader view of fatherhood exists with parental roles becoming more equivalent (Cabrera et al. 2000, 2018; Marsiglio et al. 2000; McGill 2014). The dominant conceptual model of father involvement developed by Lamb and colleagues consists of three primary components (Palkovitz 2019; Pleck 2012): positive involvement (direct interaction, e.g., caring and playing with the child), warmth and responsiveness (the emotional support offered to the child) and control (setting rules and supervising behaviour). In addition, two auxiliary domains were identified: social and material indirect care (e.g., appointments with school and the doctor) and process responsibility (monitoring and anticipating what is needed).

Recent studies interviewing professionals working with fathers in general have shown that they agree fathers have a positive impact on child development overall, but they see several barriers involving fathers and including them in parenting support (Baran and Sawrikar 2024; Brown et al. 2023; Cryer‐Coupet et al. 2021; Maxwell et al. 2012; McBride et al. 2017; Pfitzner et al. 2020; Tully et al. 2018). According to professionals who provide early interventions, fathers are often not able to join because of work, separation and custody issues or they are not engaged with parenting. In addition, service‐level barriers are prevalent, such as support being focused on mothers, professionals being often female and services keeping inflexible hours during which fathers work (McBride et al. 2017). A recent literature review on studies with both parents and professionals as respondents confirms these service‐level barriers (Baran and Sawrikar 2024). In child welfare and child protection services, similar barriers are experienced by professionals. They tend to focus on mothers, exclude fathers and label them as either good or bad. In addition, mothers often do not provide information about the fathers, and fathers are reluctant to engage in support because they fear failure or interference by services (Maxwell et al. 2012).

Studies on fathers in general have also shown several facilitators increasing the involvement of fathers in parenting support. To begin with, fathers should be identified early and involved from the start, and services should be available and accessible to all fathers by offering support at flexible opening hours and hiring male professionals (Baran and Sawrikar 2024; Maxwell et al. 2012; McBride et al. 2017). In addition, support should be tailored to fathers and should address their needs, such as support in finding work (Maxwell et al. 2012). Furthermore, involvement of fathers is increased by professional experience, competence, training and organisational support (Cryer‐Coupet et al. 2021; Tully et al. 2018). It also helps when professionals have positive attitudes towards working with fathers are actively approaching fathers (Baran and Sawrikar 2024; Maxwell et al. 2012), are passionate about fatherhood, use their personal and professional experiences with fathers, make an emotional connection by self‐disclosure, respect fathers and use shared decision making (Cryer‐Coupet et al. 2021). Finally, peer support groups in which fathers felt safe to share their parenting experiences support father engagement (Brown et al. 2023; Pfitzner et al. 2020).

So far, only a few qualitative studies with small sample sizes have been conducted on the paternal role of fathers with intellectual disabilities. Fathers with intellectual disabilities execute their paternal role in various ways: giving direct care or playing (positive involvement), being their child's teacher (control), being the head of the family and financial provider (social and material indirect care) and by supporting their partner with domestic work (More and Tarleton 2021; Symonds et al. 2020). In contrast, most mothers with intellectual disabilities think their major role as a parent is being the primary caregiver and providing physical closeness as well as warmth and affection (More and Tarleton 2021).

Fathers with intellectual disabilities wish the best for their children and want to become good parents, although some have experienced inadequate parental practices and a lack of a positive role model in their own youth (Ćwirynkało 2022; Ćwirynkało et al. 2022; Symonds et al. 2020). Nevertheless, they often face challenges in fulfilling their paternal role due to mental health issues, a problematic relationship with the other parent and inadequate parenting support from professionals (Ćwirynkało et al. 2023; Symonds et al. 2020). Especially fathers with intellectual disabilities who are involved in child welfare and child protection services often experience exclusion from parenting support, feel neglected, rejected and discriminated by professionals. They often experience a lack of control, perceive high levels of stress and despair and feel they have to prove they are good enough parents and have to fight to execute their parental role (Ćwirynkało and Parchomiuk 2023a, 2023b; Pytlowana and Stenfert Kroese 2021; Theodore et al. 2018). In addition, some fathers with intellectual disabilities feel let down by service providers; they do not receive the support they need and which was agreed upon and feel parenting support is focused on mothers (More and Tarleton 2021; Symonds et al. 2020; Theodore et al. 2018).

To our knowledge, only one study has examined facilitators in involving fathers of families of parents with intellectual disabilities in parenting support. In a study by Symonds (2020) on recruitment of parents with intellectual disabilities for parenting programmes, professionals' first contacts with parents to invite them for a parenting programme were analysed. Results showed the professionals' way of conversation may influence the engagement of both mother and father. When the relevance of the other parent was highlighted and the name of that parent was mentioned, the invitation was more likely to be accepted.

Although a few studies have been conducted on the parenting role of fathers with intellectual disabilities and the involvement in parenting support, knowledge is still lacking on how their involvement can be increased. Therefore, the current study examined (1) the views of fathers of families headed by parents with intellectual disabilities about their paternal role, and (2) barriers and facilitators according to these fathers and professionals in fulfilling their paternal role.

## 2 Methods

### 2.1 Participants

A total of nine fathers from families headed by parents with intellectual disabilities and 14 professionals took part in this study. The families received support from organisations offering specialised care to parents with intellectual disabilities, either mandated or voluntary, in the Netherlands. In families who are supported by these social services, either the mother has intellectual disabilities, or the father or both. In this study, six fathers had intellectual disabilities themselves. Three fathers did not have intellectual disabilities, but their (ex‐)partner did. They were between 21 and 49 years old and had one (*N* = 4), two (*N* = 4) or four (*N* = 1) children between 7 months and 20 years old. Most of the fathers had a full‐time job. Professionals who participated in the study were between the ages of 25 and 56 and worked at a variety of social service organisations. Four of them were male. See Tables 1 and 2 for information on living‐ and work‐situation.

**TABLE 1:** Information on living and work situation of fathers.

	*N*
Marital status	
Together with mother of child	4
Not together with mother of child	5
Living arrangements	
With child(ren)	2
With partner and child(ren)	2
Without child and mother of child	5
Housing arrangements	
Independent	4
In facility of care organisation	5
Employment	
Unemployed	1
Apprenticeship	1
Part‐time employed	1
Full‐time employed	6

**TABLE 2:** Information on work experience professionals.

	*N*
Function	
Youth protection worker	4
Family support worker	7
Personal support worker	2
Parenting consultant	1
Social service organisation	
Parenting support	2
Homeless care	1
In home parenting support	1
In home support for parents with intellectual disabilities	6
Child protection services (mandated care)	4
Relevant working experience	
0–2 years	2
2–5 years	2
5–10 years	3
> 10 years	7

### 2.2 Procedure

The study procedure was approved by the Ethics Review Board of the Faculty of Social and Behavioural Sciences, University of Amsterdam. All participants were given written and verbal information about the study and its aim. They signed an informed consent form, which was presented in an accessible and easy‐to‐understand format, in order for all participants to fully understand the scope and aim of the research.

Participants were recruited by organisations providing care for individuals and families with intellectual disabilities. Seventeen organisations were contacted and asked to distribute a flyer, which requested professionals working with fathers with intellectual disabilities to recruit fathers in their caseload who met the inclusion criteria (see below). Seven organisations recruited fathers and professionals. Fathers were provided with easy read materials and consent forms. If the fathers agreed, the interview was scheduled either through the professional or through direct contact between the father and researcher. Participating fathers were selected based on the following criteria: a suspected or diagnosed intellectual disability in one of the parents based on casefile information and professional judgement, communication skills sufficient to take part in an interview and having at least one child within the age range of 0–18 years old. Twelve fathers agreed to participate, but eventually, three fathers were no longer able to participate. A second flyer requested professionals to participate. Participating professionals were selected based on the criteria of having experience in working with fathers of families with intellectual disabilities. Fifteen professionals agreed to participate; one participant cancelled at the last minute due to personal circumstances. They did not know the participating fathers. All participants received a gift card of choice of €25 an hour. Both fathers and professionals were asked whether they wanted to participate in a focus group or in an individual interview, to adapt to their wishes and possibilities.

Interviews were conducted by three interviewers. To ensure interviews were administered in a similar way, all interviewers conducted joint interviews at the onset. Six individual interviews with fathers and professionals were conducted by one researcher, three fathers and four professionals by a duo and four professionals in a focus group with two researchers. The interviews lasted 1 h on average, with a range of 40–90 min, and the focus group 2 h. At the start of the interviews and focus group, interviewers and interviewees agreed not to mention names. All fathers were visited in person at locations of the participants' choice, such as their private homes or at their workplace, except for one father who was interviewed online. During two interviews, a professional or partner supporting the father was present at the onset of the interview and was also able to provide support or care after the interview. Professionals were all interviewed online.

### 2.3 Measures

The semi‐structured interviews with fathers included open‐ended questions about their experiences with fatherhood, parenting roles, parenting support and possible barriers and facilitators in fulfilling their parenting role. The wording of the questions was adapted to persons with intellectual disabilities, and the interview guide for fathers was tested and modified based on an interview with one father. Each interview with fathers started with a positive and empowering question: ‘What are you proud of as a father?’

The semi‐structured interviews and focus group with professionals included open‐ended questions about their experiences with fathers with intellectual disabilities, their experience with working with these fathers and possible barriers and facilitators in involving fathers and for fathers to fulfil their parenting role. Each interview started with a positive and empowering question: ‘What do you like about working with fathers from families where one of the parents has intellectual disabilities?’ The focus group with professionals used the visual platform Miro. Participants were asked to write down their ideas on the themes on digital post‐its and place them on the digital board, after which these were discussed with participants.

### 2.4 Analyses

All the interviews were audio recorded and the focus group was audio and video recorded with permission of the participants. Afterwards, the interviews and focus groups were transcribed and made anonymous, after which they were coded and analysed by the second author. The coding process used Qualcoder, a free and open source software for qualitative data analyses. A framework analysis was used, which is a qualitative method to organise and analyse data using a predefined analytical framework that consists of a set of predetermined themes or categories that are derived from the research questions or objectives, and has been used in a range of ways in applied research (Gale et al. 2013; Goldsmith 2021; Spencer et al. 2003). Analyses started with deductive coding according to the topics of the interview guideline, after which inductive coding was used by assigning codes to sections of the text. Finally, within topics, data were structurally analysed. In several rounds, different codes were clustered into categories and, where needed, renamed or recategorized, which led to creating sub‐themes. These codes were discussed in the research team. Interpreting the data, frequency of the same or similar codes in transcriptions (in more than one participant) was considered important for the development of the category or theme. Nonetheless, when a code was found in only one participant but added to other codes, it was part of the development of the category or theme as well.

## 3 Results

### 3.1 Paternal Role

Concerning the first question ‘what are the views of fathers of families headed by parents with intellectual disabilities about their paternal role?’ analyses show two themes: (1) perception of parental roles and (2) fulfilling the paternal role (see Table 3 for themes and sub‐themes).

**TABLE 3:** Themes and sub‐themes per topic by fathers (F) and professionals (P).

Paternal role	Barriers	Facilitators
Perception	Problematic relationship with the other parent	Co‐parenting
Pride (F + P)	Maternal gatekeeper (P)	Mother (F + P)
Recognition (P)	Relationship (P)	Motivation father (F + P)
Disadvantage (F + P)	Fight (F + P)	
Insecurity (F + P)		
Fulfilling the paternal role	Distance between father and family	Professionals' actions and skills
Provider (F)	Living situation (F + P)	Communication (F + *P*)
Activities (F)	Custody (F + P)	Solution focused (P)
		Gain trust (F + P)
Teaching (F)	Problems (F + P)	Recognition (F + P)
Division of tasks (F + P)	Work (F + P)	Adjustment (P)
	Motivation (P)	Characteristics (F + P)
		Education (F + P)
	Lack of involvement in parenting support	
	Working hours (P)	
	Focus on mothers (F + P)	
	Mutual distrust (F + P)	
	Stigmatisation by society (F + P)	

#### 3.1.1 Perception of Parental Roles

Fathers see their paternal role as equally important as the maternal role, are very proud of their children and want to be involved in their lives. Professionals point out that fathers want to be taken seriously as a father and in their paternal role. However, it is not uncommon that they subordinate themselves to mothers. According to both fathers and professionals, fathers believe the emotional bond between mother and child cannot be surpassed, as they have carried it during pregnancy and breastfed it. Some fathers also feel insecure in what to do with a baby in the postnatal phase, and they often let the mother take a bigger role in caring and parenting.

I think, mothers have an emotional bond with the children, also because they carried and breastfed for 9 months. But fathers also have a bond with their children, a different bond, because it's their child (Father 2).

#### 3.1.2 Fulfilling the Paternal Role

Fathers also talked about how they fulfil their paternal role, and three major themes emerged. First, they feel responsible for the family in providing for them. Therefore, work is important to them. Second, they find it important to be there for their child, give emotional support and do activities with their children. Third, they want to teach children to take care of themselves, for instance in personal care and self‐defence. All fathers said their own father was not a positive role model, which influenced their views of their paternal role.

I want to give my children what I never had myself (Father 4).

Most fathers mentioned they equally participate in parenting tasks in general, but due to a more traditional division of roles with fathers working and mothers staying at home, mothers often are responsible for the caring and domestic tasks and fathers for the income. According to professionals, paternal involvement is mainly reflected in doing games and activities outside the home, bringing their child to sports activities, having fun and being present at meetings about the child, rather than in caring tasks. However, when mothers are not capable in caring, fathers take on more of the parenting and caring tasks.

### 3.2 Barriers and Facilitators

Concerning the second question, ‘what are barriers and facilitators according to these fathers and professionals in fulfilling their paternal role?’ analyses show the following themes for barriers: (1) relationship with other parent, (2) distance between father and family, (3) involvement in parenting support, (4) stigmatisation by society, and for facilitators: (1) co‐parenting and (2) professionals' actions and skills (see Table 3 for themes and sub‐themes).

#### 3.2.1 Barriers

##### 3.2.1.1 Problematic Relationship With the Other Parent

Fathers who were separated from their partner believe that after a breakup, mothers keep the children from them and speak ill of them.

She keeps holding everything back and saying I'm a bad father. And yes, I have proved the opposite a long time ago now, with [social service provider] there present as well. And [social service provider] also speaks very highly of me, how I deal with the children (Father 12).

Professionals mentioned that mothers may indeed present a negative image of fathers in case of serious problems in the relationship and a complex separation with parental fighting. In addition mothers keep them at a distance because of previous domestic violence or the fear of interference by the new partner of their ex‐partner.

According to professionals, fathers are more likely to give up fighting for the sake of their child and distance themselves. When ex‐partners do not want to see each other anymore it is hard to stay involved. In general, according to professionals, the mother does not always give the father space to be involved in caring for and parenting their child.

More and more I notice that these vulnerable parents find it very difficult to let go and to let someone else do it. […] a lot of fathers who, at a young age for example, were still alone with their child, and then I think: ‘they are doing fine and they like it and they are good at it and they are very caring and they can do it’. My experience is also that, especially at that young age, they just get less space from the mother. Father […], just gets less space with the young child to be a father, to experiment a bit and be a bit wilder, whereas later in life […] they get much more space to also just be allowed to do things without something depends on it (Professional 6).

##### 3.2.1.2 Distance Between the Father and Family

First, both fathers and professionals indicated fulfilling the paternal role is negatively affected when fathers live separate from their family. For instance, fathers get to see the children less often than mothers, as children are often placed with their mothers in case of separation, or when mother and child live somewhere else due to housing problems.

These parents lived separately, close to each other. Father was still in a battle to get custody. Well, that did eventually work out. But actually at the time that, […] he almost got custody, mother said: ‘I'm moving to my new partner in another city’. And this man of course was completely heartbroken, saw his co‐parenting shattered, because he was not in a position to take his child to school during the week, for example, so he was demoted to a weekend father … (Professional 4).

In that mother–child house, I didn't always like that. […] Look, I do have to work 8 h a day. After that, in my free time I do want to be with my [family]. I couldn't with the first [child]. Those two days that I was off, I was with them from morning until 9 PM. I knew at 9 PM, then they really came knocking on the door, saying ‘You really have to go’. That did bother me (Father 5).

Second, fathers and professionals mentioned fathers' personal problems, such as hospitalisation in a (mental health) institution, drug abuse or imprisonment, which make it hard to be involved.

I come from a very complicated situation, so first they want to help me here to make myself better. But at the same time they slow me down to make myself better. Because I want to be with my girlfriend as much as possible, but I'm tied to the rules of this place, so … (Father 4).

They live here of course also under supervision, and sometimes they find that very difficult: ‘How can I be there for my child if I need to support myself’ (Professional 10).

Moreover, both fathers and professionals mentioned it is difficult to feel like a father (again) and to become involved (again) after being absent for a period of time. Third, due to work fathers can spend less time with their children and have a less prominent role in parenting than mothers.

I just work at [restaurant] as a chef. A full‐time job, so [it is] just kind of busy for me, 5 days and then a weekend of kids (Father 6).

##### 3.2.1.3 Lack of Involvement in Parenting Support

First, according to both fathers and professionals, social services are focused on mothers. Mothers receive support and help with parenting. Social services, such as a training home for mother and child, are not accessible enough for fathers or have strict rules, impeding them to have contact with their child and to gain parenting skills.

In the mother–child home we were mainly focused on mothers. And in some cases a father was in the picture, but our counselling focuses mainly on the mother. And then the father would visit. But in the beginning we wanted to observe how the mother was doing with the child. And then we actually wanted fathers to be in the picture as little as possible because they were often the ones who took over [parenting], so we could not observe properly. Some mothers also said ‘you know, you are actually tearing our family apart this way’ (Professional 1).

Second, according to professionals, the mother is usually the primary contact for professionals. It is not always clear to professionals who the father is, and whether the mother is still together with the father or not, which makes it hard for professionals to involve fathers in the parenting support. In addition, mothers often find it difficult to welcome the father into the support process. Especially when child protection services are involved, mothers have a stronger need to be in control.

[…] often families with a family supervision order, where people are watching, where parenting is under pressure, where they give the feeling ‘I am vulnerable, people are watching’. And then I think the mother also prefers to do it herself anyway, in her own way (Professional 6).

Third, mutual distrust exists between fathers and professionals. Fathers feel they are frequently seen as the bad guy or perpetrator and fear out‐of‐home placement of their child. According to fathers, mothers are more likely to be believed by (female) professionals, whereas fathers must fight with professionals for access to their children.

She has plotted that to have my daughter placed out of the home with the hope that she would be placed with her. If you just want to listen to [name ex‐girlfriend]… and I had nothing to say at all. And then [name ex‐girlfriend] came up with the shitty story that I was abusing [name daughter] and then the child welfare worker went to school and they took her out of school. I just had to prove that I didn't abuse my child (Father 7).

Professionals acknowledge that fathers have to fight harder against the stigma and the high expectations of professionals. Sometimes they fear fathers because of their rough appearance and use of foul language, anger or aggressiveness, which can be more intimidating than a mothers' expressions of frustrations.

You notice already they are kept at a distance anyway, because people find it frighting, because people mainly see the anger. But before that, there's very little recognition of that. And they also have a bit of a stigma of short fuse, quick to anger, aggression regulation problems. But underneath of course there's just a lot of vulnerability. If you don't see that or recognize that but you react mostly to what you see … and I think that's the experience they often have (Professional 6).

Moreover, professionals fear domestic violence as fathers are often accused of being the perpetrator. In addition, professionals indicate fathers are distrustful due to being insecure of their parenting skills, previous experiences of failure with social services, fear of stigma or embarrassment about needing help.

Fourth, professionals mentioned that overlapping working hours of professionals and fathers make it hard to involve working fathers in parenting support. On top of that, professionals consider connecting with fathers as an extra task and thus time consuming, especially when parents are divorced.

Work is an issue though. Fathers often work a lot and are not always able or willing to make time for counselling. Which I understand, but we are also bound by working hours. Look, I am quite flexible to come in an evening, but I also have my own family and work. In general, we don't work on the weekends (Professional 7).

At the same time, professionals reported that fathers find it difficult to cancel work for an appointment about their child, out of fear of losing their job. They often have jobs that do not allow leaving for several hours to attend a meeting. In addition, some professionals mentioned that fathers do not show up for appointments; they think some fathers have other priorities.

Fifth, legal rules and custody are not always clear to fathers and professionals.

Both fathers and professionals indicated that mothers seem to have more rights, and that children more often live with the mother after separation, while the father gets visitation rights. Few fathers have applied for joint custody when not married as they are not familiar with legal regulations and the advantages. Professionals mentioned privacy rules prevent them from informing fathers about the child's situation at mother's place after separation and they are not obligated to involve fathers who do not have custody.

##### 3.2.1.4 Stigmatisation by Society

Fathers mentioned society is focused on mothers, for instance by giving priority to mothers in searching for suitable housing, while fathers are left to find a home for their own. In addition, both fathers and professionals mentioned that many people have negative beliefs and prejudices on fathers with intellectual disabilities and on single fatherhood (they cannot parent; they are the perpetrator). They are thus doubly stigmatised by family, school and social services. This gives both fathers and professionals the idea they have to work harder to prove to professionals and society that fathers are able to care for their children.

If you are in a divorce and all kinds of things have to be arranged. There's a whole process about that, too. And yes, and that's where [name support organisation] also helps me a lot. It is just difficult to be a father in the Netherlands. I may have authority, but that is not the whole story. I mean, the woman has too many rights. So the woman, she can just decide and do and be where she wants. Because she just has too many rights here (Father 12).

#### 3.2.2 Facilitators

##### 3.2.2.1 Co‐Parenting

According to fathers and professionals, fathers get a larger role in parenting if mothers involve them, or when the mother is less able or unable to care for their children. In addition, they both mention that fathers who make an effort for involvement usually get involved.

##### 3.2.2.2 Professionals' Actions and Skills

First, fathers also want to be informed about their children and invited to meetings about their children. This gives them the feeling of recognition. Professionals agree that fathers want to and should be treated the same as mothers with regard to informing them about their child. Thus, professionals mentioned they should also establish direct contact with fathers from the start and not only through mothers. In addition, they mentioned it is important to communicate transparently, make clear agreements and stick to them.

I think that when you don't speak and you indeed start a conversation with mother and you don't include father, especially in the initial phase, for example during an initial conversation or in the first period, then I think the father quickly drops out. So I think that requires an active role of social work to involve him directly (Professional 4).

Moreover, professionals should be committed to fathers and search for possibilities to get fathers involved, and include and support them in meetings with (social) services and make sure other organisations invite and inform fathers.

Second, fathers want professionals to listen to their experiences and needs in order to feel they are seen and heard. Professionals confirm this and indicate it is important to show interest in the story of fathers, to examine emotions behind the behaviour and to highlight that the opinions and roles of fathers do matter.

Third, professionals pointed out that they should adapt to fathers' level and needs by using their language and meet them wherever fathers feel comfortable. Social service organisations should introduce flexible working hours, enabling professionals to make appointments at hours at which father can be present. Some fathers indicated they want male professionals, because they are easier to talk with and professionals with children of their own so they can give advice from their own experiences. Professionals agreed that matching fathers to male professionals may be helpful and that male professionals have more understanding for fathers' position, open up more easily, can serve as a role model and build working relationships faster.

Fourth, fathers mentioned they need advice and practical coaching on parenting skills and their paternal role, and education on developmental stages of children and on how to care for a baby. Professionals agreed and added three important tasks for themselves. First, professionals should educate both fathers and mothers about the importance of the paternal role for the development of the child and coach them in how they can work together to ensure the father can fulfil this role.

The first time, I really didn't know how to hold a baby. So small. So you must get education. I started looking on the Internet myself. Fathers don't really have that understanding of it. Or they have to get a course or have a book. But some people like to read a book, who can learn. But some still maybe should get a course (Father 5).

Second, fathers, however, need more than parenting support and want to talk about and be advised on other subjects as well, such as relationships, work or housing. Professionals confirmed talking about other things than parenting is important to establish a relationship and to gain trust. This, however, takes time and requires to be patient and available.

Third, professionals should explain the legal context of parenting and how to apply for custody. When a father has custody, professionals are obligated to involve fathers, but it should be clear to both professionals and fathers, having custody or not, what their rights and duties are.

## 4 Discussion

The aim of this study was to examine (1) the views of fathers of families headed by parents with intellectual disabilities about their paternal role, and (2) what the barriers and facilitators according to these fathers and professionals are in fulfilling their paternal role. The findings show that fathers of families headed by parents with intellectual disabilities consider their father role to be important and want to fulfil this role. They mostly understand their role to be a provider, engaging in a wide variety of activities and teaching their children (life) skills and how to function in the world. Although fathers say they participate equally in parenting tasks, the division of tasks between parents is in most cases more traditional, which is in line with previous studies on fathers with intellectual disabilities (More and Tarleton 2021; Symonds et al. 2020). Still, also in these families we see a shift and more involvement in parenting by fathers. Even though the mothers in most of the families headed by parents with intellectual disabilities may perform more of the parenting tasks, fathers do believe they are just as important as mothers; they want to be recognised and taken seriously as fathers and be involved in parenting their children in a way that is compatible with their situation. Nevertheless, the results of this study also show that fathers are hampered in fulfilling their paternal role because of several barriers at the family, professional and societal levels.

First, at the family level, because of a complex interaction between parents with intellectual disabilities, with fathers not capable of taking up their paternal role and mothers not always allowing them to do so. This pattern, also known as maternal gatekeeping (Gaunt 2008), is seen between parents in general as well, and seems to develop gradually from (pre)birth and is difficult to break. In addition, results showed that when severe relationship problems arise and parents with intellectual disabilities separate, mothers tend to keep fathers (even more) at a distance, and their legal status is often more clear, hence most often children end up staying more time with the mother. Moreover, when conflict situations get worse, these fathers also become more avoidant and sometimes disappear, because they think it is less harmful for their child. They see themselves as subordinate, also because they may have had fewer tasks in parenting, and may assume that the parent who spent the most time with the child also has more rights. Obviously, when fathers do not live with their children, and work full time, they have less contact with their children and cannot fulfil their paternal role as they may wish. These findings are not found in previous studies, but provide more insight in the complex relationship with the other parent.

Second, when social services are involved to provide families with parenting support, one would expect that there are more opportunities for paternal involvement. Professionals are, however, not inclined to involve fathers of these families in parenting support practices, which is in line with previous studies in child welfare and child protection services (Ćwirynkało and Parchomiuk 2023a, 2023b; Pytlowana and Stenfert Kroese 2021; Theodore et al. 2018). In addition, parenting support for parents with intellectual disabilities is focused on mothers, as was also found in previous studies with fathers in general (McBride et al. 2017) and fathers with intellectual disabilities (More and Tarleton 2021; Symonds et al. 2020; Theodore et al. 2018). Mothers are often the primary contact for social services, and especially when there are severe relationship problems, they may speak ill of fathers to professionals. Professionals tend to believe mothers and are usually not unbiased in the first contact with fathers. This increases the distrust and resistance of fathers as they feel they have to fight for their right as a parent. Hence, professionals have difficulties building a working relationship and may eventually avoid fathers and do not involve them in parenting support, especially when fathers have no legal custody and do not live with their children.

Third, at the societal level, fathers from families with intellectual disabilities also feel stigmatised by society, because many people have prejudices about single men with a child, and the relationship between mother and child is valued more strongly. Moreover, when fathers have intellectual disabilities they are doubly stigmatised, and hence ignored as a potentially important people in the lives of their children, as there is still a wide belief in society that parents with intellectual disabilities cannot parent (Franklin et al. 2022; Theodore et al. 2018). Fighting for their paternal role, against the prevailing stigmas, fathers may feel compelled to step back from parenting their children, even though they would prefer otherwise. Professionals may see this as a sign that fathers are less important and risky to involve.

There are, however, also opportunities to increase the fulfilment of the paternal role of fathers from families with intellectual disabilities The focus of social services and parenting support organisations should be broader, with support being adjusted to fathers' needs. This is in line with previous research on fathers in general (Maxwell et al. 2012). Especially in a changing society in which fathers are more involved in parenting and mothers are more often inclined to have jobs as well, it is no longer an option to discard the importance of fathers in parenting (Cabrera et al. 2000, 2018; Marsiglio et al. 2000; McGill 2014), to leave them out and to not assist and support them as well to be the best father they can be. In addition, increasing positive development of children requires highly committed parents and systemic working, supporting parents in parenting and addressing problems of all members of the family (Dunst et al. 2007). Considering that the intellectual disability, mental health problems and challenges in the relationships all influence the way a father can take up his paternal role, mental health support should be integrated with support on parenting and marital problems. This is in line with suggestions in previous studies on fathers with intellectual disabilities (Ćwirynkało and Parchomiuk 2023b; Symonds et al. 2020).

To work with fathers of families with intellectual disabilities, professionals need to build trust and invest in creating a working relationship. To do so, they need to establish contact with them, listen to them, take them seriously, adjust to their needs and give them a voice. This is in line with fathers in general (Cryer‐Coupet et al. 2021; Wang and Chen 2024) and is not different from working with mothers with intellectual disabilities (Koolen et al. 2020; Meppelder et al. 2014). Professionals should improve their skills in building working relationships and be aware of the difficulties for fathers to discuss issues with a female professional. In addition, they should recognise and discuss their own stigmatising beliefs with colleagues (McConnell and Phelan 2022).

Because of the way social services are organised, it may be harder to establish a relationship with fathers of families with intellectual disabilities. Professionals should work on the relationship with fathers from the start and try to involve fathers from the beginning of the pregnancy, inform and invite them, which has also been suggested in previous studies on fathers in general who are dealing with child welfare services (Baran and Sawrikar 2024; Maxwell et al. 2012; McBride et al. 2017). This study shows that professionals should also accept and understand that for fathers of families with intellectual disabilities, their job is very important and thus they are not always able to attend a meeting. Together with both parents, professionals should look for a date and time that suits them all. Furthermore, fathers are more willing than is believed. They indicate they want to find solutions and want to be involved in support, and they are willing to show up; they just do not always know how they can make it all work and may need some help in thinking of potential solutions, such as addressing the situation and asking their employer for time off.

Parents with intellectual disabilities should be supported by professionals in fulfilling their parenting role, in order to increase the well‐being for, and the positive development of, their children. Results showed that co‐parenting would be beneficial for fulfilling the paternal role of fathers of families with intellectual disabilities, which is in line with previous studies in the general population, which show that paternal involvement is related to whether mothers believe the father of their child should be involved (Fagan and Barnett 2003; McBride et al. 2005; Zvara et al. 2013) and to co‐parenting closeness (Osavsky et al. 2020). Parents with intellectual disabilities and professionals should be aware that qualities of both parents are important in parenting, that parents with intellectual disabilities tend to have a high divorce prevalence (Sterenborg et al. 2023), and thus it is important to focus on a good relationship. Mothers therefore have to be informed that the father role and involvement is important for the development of their child (Dyer et al. 2009; McBride et al. 2017) and may also relieve her from stress in parenting (Boyd et al. 2019; Hartley et al. 2011; Kanter and Proulx 2019; Malinen et al. 2017). In addition, mothers should be supported in giving space to fathers to fulfil their paternal role, in improving skills and developing language to discuss roles. Fathers should be supported in fulfilling their paternal role, next to educating them in child development and practical parenting skills.

Finally, professionals should support fathers of families with intellectual disabilities in understanding legal regulations on custody, their rights and obligations and support them in applying for legal custody. When fathers have legal custody, they have more rights and professionals are obligated to involve fathers in important decisions about their child's life. However, professionals should be well informed themselves, because they tend to think privacy rules prevent them from informing fathers or fathers without legal custody have no right on information. Even without legal custody, fathers have the right to receive information about their child, at least in the Netherlands. Thus, an important task for professionals is to support fathers in positioning and involving them in the lives and parenting of their children. Experience experts and advocacy organisations may play a role in informing fathers (Simpson and Chan 2021) by providing success stories, online information, and group sessions (cf. www.bumpyroad.org.au; Spencer and Collings 2021).

## 5 Strengths and Limitations

A strength of this study is that both professionals and fathers of families with intellectual disabilities were interviewed, not only on barriers but also on facilitators for involvement. Fathers and professionals touched on similar topics. Professionals even mentioned some more barriers and facilitators, that tied in nicely with what fathers already had mentioned providing relevant insights from different perspectives. All professionals in this study worked with fathers from families with intellectual disabilities and thus may have been more aware of barriers and facilitators for fulfilling the paternal role. Thus, these insights can be used by other professionals to increase awareness of fathers' needs and roles and stimulate and support fathers in fulfilling these. This study shows that the lack of involvement of fathers is clearly a general issue in both fathers with and without intellectual disabilities. It adds facilitator themes to previous studies on fathers with intellectual disabilities and elaborates on and explains the feelings of being disadvantaged by fathers, especially by the complex interaction with the other parent and double stigmatisation.

Nevertheless, results should be interpreted with care as this study also has some limitations. First, despite high efforts to recruit fathers, we have only included nine fathers. Fathers seemed to be hard to reach, which may be due to the recruitment strategy via organisations and professionals who, as has become clear from literature and this study, have less contact with fathers than with mothers. In addition, we have recruited fathers from organisations who support parents with intellectual disabilities. These organisations support families in which either the mother has intellectual disabilities, or the father or both. Thus, our sample reflects the Dutch situation and includes both fathers with and without intellectual disabilities. A diagnosis of intellectual disabilities of parents, however, is not always available. Thus, the sample of the present study may comprise more or fewer fathers with intellectual disabilities. Nevertheless, none of the themes has emerged from answers only from fathers with intellectual disabilities or fathers without intellectual disabilities.

Second, the sample consisted of 90% working fathers and 55% divorced fathers not living with their children, which may have influenced the results. This may have yielded the themes on the complex interaction with the other parent and the double stigmatisation. Third, the interviews with fathers were conducted live, whereas interviews with professionals were online and in one focus group. These different settings may have caused that professionals were not heard equally, but at least, fathers were spoken extensively.

## 6 Future Research

Future research should include more fathers from different backgrounds, such as fathers from different nationalities, and focus on differences in barriers and facilitators between fathers who live separated from their partners and fathers who live at the same home or working versus non‐working fathers. Differentiating between groups increases knowledge on how support can best be adjusted to the specific needs of fathers. In addition, once barriers and facilitators for father involvement are known, tools and methods should be developed and implemented in practice.

To conclude, this study has pointed out that fathers of families headed by parents with intellectual disabilities want to be involved and recognised as a parent and to fulfil their paternal role. Several barriers, however, hamper fulfilling their paternal role and involving fathers in parenting support. Increasing attention for fathers is needed in society and social services. Therefore, the needs, possibilities and wishes of fathers should be heard. Professionals should be open to them, ask questions, listen to them and together examine what these fathers need to be the best fathers for their children. We should implement facilitators in parenting support in order to enable both father and mother to fulfil their parental role and to improve parental rights and development and well‐being of their children.

## Ethics Statement

The study procedure has been approved by the Ethics Review Board of the University of Amsterdam (case number: 2022‐CDE‐14368).

## Conflicts of Interest

The authors declare no conflicts of interest.

## Data Availability

The data that support the findings of this study are available on request from the corresponding author. The data are not publicly available due to privacy or ethical restrictions.
